# Adaptation and Validation of the Social Media Cyberbullying Victimization Scale Among Chinese College Students: Cross-Sectional Study

**DOI:** 10.2196/88857

**Published:** 2026-06-11

**Authors:** Chen Xu, Suping Wang, Yujie Liu, Xin Ge, Xue Yang, Xiaohong Fan, Li Li, Ying Wang, Yong Cai

**Affiliations:** 1 Institute of Clinical Research, Tongren Hospital, Shanghai Jiao Tong University School of Medicine, No. 1111, Xianxia Road, Changning District, Shanghai, 200336, China; 2 School of Public Health, Shanghai Jiao Tong University School of Medicine, Shanghai, China; 3 JC School of Public Health and Primary Care, Faculty of Medicine, The Chinese University of Hong Kong, Hong Kong, China; 4 Shanghai Public Health Clinical Center, Fudan University, Shanghai, China; 5 Department of Nursing, Shanghai Mental Health Center, Shanghai Jiao Tong University School of Medicine, Shanghai, China; 6 Center for Community Health Care, China Hospital Development Institute, Shanghai Jiao Tong University, Shanghai, China

**Keywords:** cyberbullying victimization, social media, psychometric properties, mental health, college students

## Abstract

**Background:**

Cyberbullying victimization is a significant risk factor for poor psychological well-being among college students. Existing tools fail to capture the distinct dimensions of victimization in social media contexts.

**Objective:**

This study aimed to adapt and validate a Social Media Cyberbullying Victimization Scale (SMCVS) and examine its associations with psychological outcomes among Chinese college students.

**Methods:**

In Shanghai, China, 1766 students from multiple universities completed questionnaires including demographic information, the SMCVS, the Patient Health Questionnaire-9, and the Generalized Anxiety Disorder-7 scale. The psychometric evaluation of the SMCVS included content validity, construct validity, criterion validity, internal consistency reliability, split-half reliability, and test-retest reliability. Receiver operating characteristic analyses were used to evaluate predictive ability for depression and anxiety.

**Results:**

Content validity was satisfactory, and 13 items were retained, with item-level content validity index values ranging from 0.833 to 1 and a scale-level content validity index of 0.949. Exploratory factor analysis identified 2 dimensions—“information harassment” and “reputation and privacy violation”—explaining 82.2% of the total variance. Confirmatory factor analysis supported this structure (goodness-of-fit index=0.943, comparative fit index [CFI]=0.982, normed fit index=0.978, and root mean square error of approximation [RMSEA]=0.075). Multigroup confirmatory factor analysis supported configural and metric invariance across both gender and academic major (ΔCFI≤0.01, ΔRMSEA≤0.015); scalar invariance was achieved for gender but not for academic major. Both dimensions showed significant correlations with depression (*r*=0.433‐0.457) and anxiety (*r*=0.356‐0.372). Cronbach α, Spearman-Brown coefficient, and test-retest reliability coefficient were 0.959, 0.973, and 0.860, respectively. The SMCVS demonstrated moderate discriminative accuracy for depression (area under the curve [AUC]=0.738; optimal cutoff=19.5) and anxiety (AUC=0.739; optimal cutoff=22.5).

**Conclusions:**

The SMCVS demonstrates sound psychometric properties and may be useful for assessing social media cyberbullying victimization among Chinese college students. Its validated 2D structure clarifies distinct patterns of victimization and their psychological correlates, offering implications for targeted prevention and intervention.

## Introduction

Cyberbullying has evolved dramatically with the widespread use of social media platforms in the current digital age and has emerged as a critical global public health challenge [1]. Cyberbullying refers to deliberate and aggressive behavior by an individual or a group, conducted through electronic means of communication, occurring repeatedly over time, and targeting victims who struggle to defend themselves [2]. Involvement in cyberbullying can occur in 3 forms: being a victim, engaging as a perpetrator, or witnessing it as a bystander [3]. There is a broad consensus on 3 defining elements of cyberbullying: harmfulness, repetitiveness, and a power imbalance between bullies and victims [4]. Experiencing cyberbullying victimization (CV) on social media has been associated with numerous adverse outcomes, including heightened psychological distress and physical health problems [5], elevated symptoms of depression and anxiety [6], lower levels of life satisfaction [7], and suicidal thoughts and attempts [8]. These findings underscore the urgent need to address cyberbullying as a significant threat to mental and physical health worldwide.

University students, as digital natives and intensive social media users, constitute a particularly vulnerable population for CV. Although cyberbullying among middle and high school students has been extensively studied, university students have received relatively limited attention, leaving a critical gap in our understanding of this issue in this population. Previous studies have reported that the overall prevalence of CV among university students ranges from 19% to 24.1% [9,10]. Furthermore, existing research consistently highlights strong associations between CV and adverse psychological outcomes, particularly depression and anxiety, which are major contributors to the global burden of disease among young adults. For instance, a 1-unit increase in the CV score has been associated with a 1.2-fold higher likelihood of experiencing depression and anxiety [11]. These findings underscore the pressing need for further research to elucidate the unique characteristics and impacts of CV in university student populations.

The unique features of social media have amplified the scope and complexity of cyberbullying compared with traditional forms. Unlike traditional bullying, social media–based victimization exhibits characteristics that often transcend cultural boundaries, including persistent digital footprints, rapid cross-platform dissemination, and the potential for complete anonymity. These characteristics have fundamentally transformed the nature and impact of victimization experiences [12]. Cyberbullying involves the use of social media platforms, as well as fake profiles and identities, to harass, intimidate, or target others. Such behaviors commonly include disseminating threats and rumors, exposing private information, and promoting social exclusion or isolation [13]. Based on the nature of the aggressive behaviors, cyberbullying can be categorized into several types, such as flaming (also referred to as roasting), harassment, denigration, defamation, outing, trickery, sexual harassment, and cyberstalking [14]. International research has begun to identify distinct patterns of cyberbullying and to develop diverse instruments specifically tailored to social media contexts. A review of cyberbullying measurement tools over the past two decades identified 25 studies covering 17 distinct instruments [15], with only one study conducted among Chinese adolescents [16], underscoring the scarcity of culturally specific research in the Chinese context.

Self-report scales are among the most important tools for assessing and identifying cyberbullying and CV. Currently, researchers commonly use two approaches to measure these phenomena: (1) using a single-item question to inquire about individuals’ experiences of cyberbullying or CV within a specified time frame and (2) administering multi-item instruments that assess specific behaviors related to cyberbullying and/or CV [17,18]. The former approach has been shown to underestimate the prevalence of cyberbullying and CV [19], whereas the latter lacks consensus regarding which behaviors should be considered as constituting cyberbullying and CV [20]. In many cases, the psychometric properties of cyberbullying and CV scales are not rigorously evaluated before use. For example, a review by Berne et al [21] reported that fewer than half of the 44 included studies provided information on internal consistency. This lack of methodological consistency makes it challenging to compare findings across studies. To address issues of measurement consistency and validity, researchers have called for the development of theoretically and empirically grounded instruments to systematically assess cyberbullying and CV.

Therefore, this study aimed to adapt and validate the Social Media Cyberbullying Victimization Scale (SMCVS), thereby contributing to the global toolkit for assessing CV. We hypothesized that (1) social media cyberbullying victimization, as measured by the SMCVS, would exhibit a clear multidimensional structure; (2) the resulting dimensions would demonstrate acceptable psychometric properties across validation analyses; and (3) these dimensions would show significant associations with symptoms of depression and anxiety.

## Methods

### Participants

A cross-sectional study was conducted among university students in Shanghai, China, from September to December 2024. A stratified cluster random sampling method was adopted, with 5 universities serving as the sampling frame. Participants were stratified by academic discipline (medical sciences, science and engineering, and social sciences) and academic year (freshman to senior). Within each university, student groups (classes) were defined as clusters according to discipline and academic year. Subsequently, several clusters were randomly selected within each stratum, and all students in the selected clusters were invited to participate in the survey. The inclusion criteria were as follows: (1) full-time undergraduate students; (2) active social media users, defined as using social media platforms at least once per day; and (3) voluntary participation with informed consent.

### Recruitment and Data Collection Procedures

In the preliminary stage of this study, a research team was established, consisting of the principal investigator and graduate student investigators. The team developed an investigator manual and conducted 2 systematic training sessions. The training covered the study objectives, ethical requirements, operational procedures, frequently asked questions, and emergency response plans. Before the formal survey, a pilot study involving 30 medical students was conducted to assess the average questionnaire completion time (approximately 25 minutes) and to refine the questionnaire’s layout and instructions based on participant feedback.

Following the sampling design, systematic recruitment procedures were implemented to ensure standardized data collection. Survey notifications and approvals were first coordinated through the student affairs departments. Recruitment primarily took place in designated class meetings. University counselors assisted the research team by introducing the study to the students and distributing the survey via QR codes. An informed consent form was presented on the first page of the online survey, clearly outlining the study purpose, the voluntary nature of participation, and privacy protection measures.

The professional version of the Wenjuanxing platform was used for questionnaire design and data collection. To facilitate systematic tracking across the strata, separate QR code links were generated for different grades and majors. During the data collection sessions, trained investigators were physically present (on-site) in the classrooms to provide guidance and address any immediate questions from the students, while 2 dedicated staff members handled any online inquiries. To ensure data quality, IP restrictions were implemented so that each device could complete the survey only once, and the acceptable completion time was limited to 15‐30 minutes. Mandatory response settings were applied to prevent missing critical information, and one logic-check item was included to monitor response attentiveness. The status of questionnaire returns was monitored daily, and any anomalies were addressed promptly.

### Ethical Considerations

The study protocol was approved by the Ethics Committee of Shanghai Jiao Tong University School of Medicine (no. SJUPN201907). Electronic informed consent was obtained from all participants prior to their inclusion in the study. To ensure privacy and confidentiality, all collected data were strictly anonymized, and no personally identifiable information was collected. Student participation was entirely voluntary, and therefore, no financial compensation was provided. Psychological counseling resources were made available, and high-risk cases identified during the screening process were promptly referred for further evaluation and intervention.

### Measures

#### Demographic Characteristics

Demographic variables were collected, including age, gender, academic major, only child, family economic status, parents’ marital status, father’s education, mother’s education, academic performance, academic stress, smoking, and drinking.

#### Social Media Cyberbullying Victimization Scale

The SMCVS used in this study can be traced back to the Facebook-related CV scale originally developed by Kwan and Skoric [22], in which all items referred specifically to cyberbullying experiences on Facebook. This instrument was subsequently adapted into a Chinese version by Chen Qiyu and colleagues [23], who replaced the word “Facebook” in the item wording with “social network sites” to assess Chinese adolescents’ CV experiences. Later, Li [24] adopted this Chinese CV scale and further revised the wording to better fit the linguistic habits of secondary vocational students. However, neither of these two Chinese versions reported a systematic psychometric evaluation of the scale. This study adopted the Chinese CV scale adapted by Li [24] as the SMCVS and conducted the first comprehensive psychometric evaluation among Chinese college students. The SMCVS contained 17 items assessing several forms of CV, including verbal insults and harassment, denigration, impersonation, deception, social exclusion, and cyberstalking. Items were rated on a 6-point Likert scale, where 1 indicated “never,” 2 indicated “once,” 3 indicated “2‐4 times,” 4 indicated “5‐7 times,” 5 indicated “8‐10 times,” and 6 indicated “more than 10 times.” Higher total scores reflected more frequent experiences of CV.

#### Psychological Well-Being Measures

Depressive symptoms were assessed using the Patient Health Questionnaire-9 (PHQ-9), a 9-item self-report instrument commonly used in nonpsychiatric settings [25]. Participants rated the severity of their symptoms over the past 2 weeks on a scale from 0 (“not at all”) to 3 (“nearly every day”). Total scores range from 0 to 27, with a threshold of 10 or above recommended to indicate clinically significant depressive symptoms [26]. Previous studies have demonstrated the reliability and validity of the Chinese version of the PHQ-9 [27]. In this study, the scale demonstrated excellent internal consistency, with a Cronbach α of 0.934.

Anxiety symptoms were assessed using the Generalized Anxiety Disorder-7 (GAD-7) scale, which comprises 7 items designed to evaluate how frequently individuals experienced anxiety symptoms over the past 2 weeks [28]. Response options included “not at all,” “several days,” “more than half the days,” and “nearly every day,” with scores assigned from 0 to 3, respectively. Total scores range from 0 to 21, with higher scores indicating greater anxiety symptom severity. A cutoff score of 10 was used to identify likely cases of generalized anxiety disorder. The GAD-7 has been validated in China as a reliable instrument for screening and assessing anxiety symptom severity [29,30]. In this study, the scale demonstrated excellent internal consistency, with a Cronbach α of 0.947.

### Statistical Analysis

Data were presented as mean (SD) for normally distributed continuous variables and as median (IQR) for nonnormally distributed variables. Categorical variables were summarized using frequencies and percentages. Content validity of the initial SMCVS was assessed prior to the main psychometric analyses. A panel of 6 experts in the relevant field independently rated the relevance of each item. Item-level content validity indices (I-CVI) and the scale-level content validity index/average (S-CVI/Ave) were computed as the proportion of experts rating an item as relevant. Item analysis of the SMCVS included descriptive statistics, corrected item-total correlations, and Cronbach α if item deleted for all 13 items.

To evaluate construct validity, the total sample (n=1766) was randomly split into two subgroups for exploratory factor analysis (EFA; n=883) and confirmatory factor analysis (CFA; n=883). Chi-square tests were performed to compare demographic differences between the two groups. The suitability for factor analysis in the EFA subsample was assessed using the Kaiser-Meyer-Olkin test and Bartlett test of sphericity. Principal component analysis with Promax rotation was used to extract common factors, retaining components with eigenvalues greater than 1. Items with factor loadings below 0.4 and/or high cross-loadings were considered for removal or further investigation [31]. CFA was conducted using maximum likelihood estimation. Model fit was evaluated using multiple indices: chi-square goodness of fit (*χ*²/*df*), goodness-of-fit index (GFI), comparative fit index (CFI), normed fit index (NFI), Tucker-Lewis index (TLI), incremental fit index (IFI), root mean square error of approximation (RMSEA), and standardized root mean square residual (SRMR). Acceptable model fit was defined as *χ*²/*df*<5, GFI, CFI, NFI, TLI, and IFI>0.9, RMSEA<0.08, and SRMR<0.05 [32].

Measurement invariance of the SMCVS across gender and academic major was examined using multigroup CFA. For each grouping variable, a sequence of configural, metric, and scalar models was tested. Model fit was evaluated using *χ*²/*df*, CFI, and RMSEA, and changes between nested models were assessed with ΔCFI and ΔRMSEA. Values of ΔCFI≤0.01 and ΔRMSEA≤0.015 were taken as evidence of acceptable invariance across groups [33,34]. The criterion validity of the SMCVS was assessed using the PHQ-9 and GAD-7 as external criterion measures. Reliability was assessed through internal consistency reliability (Cronbach α coefficient), split-half reliability (Spearman-Brown coefficient), and test-retest reliability (intraclass correlation coefficient and correlation coefficient).

Receiver operating characteristic (ROC) curve analyses and the area under the curve (AUC) were used to evaluate the predictive ability of the SMCVS total and subscale scores for depression and anxiety, using established clinical cutoff points for the PHQ-9 and GAD-7 as external criteria. For each outcome, the optimal cutoff score for the SMCVS was determined by maximizing the Youden index (sensitivity + specificity − 1), and the corresponding sensitivity, specificity, positive likelihood ratio (LR+), and negative likelihood ratio (LR−) were calculated. All analyses were performed using SPSS 26.0, Amos 26.0, and R (version 4.3.2; *pROC* package for ROC analyses), with statistical significance set at *P*<.05 (2-tailed).

## Results

### Descriptive Analysis

A total of 1766 valid questionnaires were collected, yielding a response rate of 92.4%. The sample included 660 participants from Shanghai Jiao Tong University School of Medicine, 473 from Shanghai Sanda University, 252 from the University of Shanghai for Science and Technology, 248 from Shanghai University of Political Science and Law, and 133 from Shanghai University of Traditional Chinese Medicine.

The demographic characteristics of the participants are presented in Table 1. Participants were aged 18‐24 years, with a mean age of 19.87 (SD 1.52) years. The participants consisted of 857 (48.5%) males and 909 (51.5%) females. Nearly half of the participants were enrolled in medical-related disciplines (n=793, 44.9%) and were only children (n=885, 50.1%). Most participants (n=1599, 90.5%) reported that their parents were married. The majority (n=1156, 65.5%) reported a medium family economic status. In addition, 45.5% (n=803) of fathers and 39.4% (n=695) of mothers had attained at least a college-level education. Overall, 37.4% (n=660) of participants reported good academic performance and 51.5% (n=910) reported moderate levels of academic stress. Furthermore, 7.1% (n=125) of participants reported smoking behavior and 24.8% (n=438) reported alcohol consumption.

**Table 1.** Demographic characteristics of college students (n=1766).

Characteristics	Total (n=1766), n (%)	EFA^a^ (n=883), n (%)	CFA^b^ (n=883), n (%)	Chi-square (*df*)	*P* value
Age (years)				7.1 (3)	.07
18	339 (19.2)	176 (19.9)	163 (18.5)		
19	500 (28.3)	227 (25.7)	273 (30.9)		
20	398 (22.5)	198 (22.4)	200 (22.7)		
≥21	529 (30.0)	282 (31.9)	247 (28.0)		
Sex				0.1 (1)	.74
Male	857 (48.5)	425 (48.1)	432 (48.9)		
Female	909 (51.5)	458 (51.9)	451 (51.1)		
Academic major				0.3 (2)	.86
Medical science	793 (44.9)	393 (44.5)	400 (45.3)		
Science and engineering	252 (14.3)	124 (14.0)	128 (14.5)		
Social science	721 (40.8)	366 (41.4)	355 (40.2)		
Only child				0.1 (1)	.74
Yes	885 (50.1)	439 (49.7)	446 (50.5)		
No	881(49.9)	444 (50.3)	437 (49.5)		
Family economic status				0.9 (2)	.63
Low	203 (11.5)	102 (11.6)	101 (11.4)		
Medium	1156 (65.5)	586 (66.4)	570 (64.6)		
High	407 (23.0)	195 (22.1)	212 (24.0)		
Parents’ marital status				1.1 (1)	.29
Married	1599 (90.5)	793 (89.8)	806 (91.3)		
Divorced	167 (9.5)	90 (10.2)	77 (8.7)		
Father’s education				4.5 (2)	.10
≤Junior high	567 (32.1)	304 (34.4)	263 (29.8)		
Senior high	396 (22.4)	188 (21.3)	208 (23.6)		
≥College	803 (45.5)	391 (44.3)	412 (46.7)		
Mother’s education				1.8 (2)	.41
≤Junior high	655 (37.1)	339 (38.4)	316 (35.8)		
Senior high	416 (23.6)	198 (22.4)	218 (24.7)		
≥College	695 (39.4)	346 (39.2)	349 (39.5)		
Academic performance				1.4 (2)	.49
Good	660 (37.4)	342 (38.7)	318 (36.0)		
Average	813 (46.0)	399 (45.2)	414 (46.9)		
Poor	293 (16.6)	142 (16.1)	151 (17.1)		
Academic stress				0.5 (2)	.77
Low	287 (16.3)	138 (15.6)	149 (16.9)		
Medium	910 (51.5)	460 (52.1)	450 (51.0)		
High	569 (32.2)	285 (32.3)	284 (32.2)		
Smoking				1.0 (1)	.31
Yes	125 (7.1)	68 (7.7)	57 (6.5)		
No	1641 (92.9)	815 (92.3)	826 (93.5)		
Drinking				2.4 (1)	.12
Yes	438 (24.8)	233 (26.4)	205 (23.2)		
No	1328 (75.2)	650 (73.6)	678 (76.8)		

a EFA: exploratory factor analysis.

b CFA: confirmatory factor analysis.

### Content Validity

Content validity of the initial 17-item SMCVS was evaluated by a panel of 6 experts in the field, who rated the relevance of each item. In line with commonly recommended standards for the I-CVI ≥0.78 [35], items meeting this criterion were retained and items below this threshold were deleted, resulting in a 13-item version of the SMCVS. The wording of several retained items was slightly revised to improve clarity and alignment with the target population. For these 13 items, the I-CVI values ranged from 0.833 to 1, and the S-CVI/Ave was 0.949, indicating acceptable overall content validity.

### Item Analysis

Table 2 presents the descriptive statistics, corrected item-total correlations, and Cronbach α if item deleted for all 13 items of the SMCVS. The mean scores for the items ranged from 1.41 to 3.01, with SDs between 1 and 1.53. Corrected item-total correlations ranged from 0.385 to 0.897, indicating moderate to strong item-total relationships. Notably, item 1 demonstrated the lowest corrected item-total correlation (*r*=0.385), whereas items 7 and 8 showed the highest correlations (*r*=0.897). The Cronbach α values if item deleted ranged from 0.953 to 0.969. The internal consistency of the scale would not be substantially improved by removing any single item.

**Table 2.** Descriptive statistics and item analysis of the Social Media Cyberbullying Victimization Scale (n=1766).

Items	Mean (SD)	Corrected item-total correlation (*r*)	Cronbach α if item deleted
Item 1	3.01 (1.53)	0.385	0.969
Item 2	2.05 (1.41)	0.691	0.959
Item 3	1.72 (1.27)	0.808	0.955
Item 4	1.86 (1.35)	0.689	0.959
Item 5	1.55 (1.08)	0.888	0.953
Item 6	1.53 (1.08)	0.894	0.953
Item 7	1.53 (1.07)	0.897	0.953
Item 8	1.52 (1.08)	0.897	0.953
Item 9	1.54 (1.07)	0.859	0.954
Item 10	1.48 (1.04)	0.885	0.953
Item 11	1.45 (1.01)	0.888	0.953
Item 12	1.41 (1.00)	0.887	0.953
Item 13	1.45 (1.00)	0.877	0.954

### Construct Validity

#### Exploratory Factor Analysis

**Figure 1.**
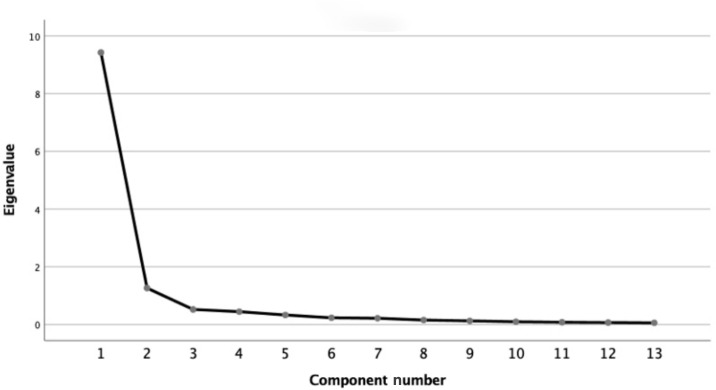
Scree plot from exploratory factor analysis of the 13-item Social Media Cyberbullying Victimization Scale (n=883). Eigenvalues are plotted against component numbers, showing a clear inflection after the second component and supporting a 2-factor solution.

The demographic characteristics of the EFA and CFA subsamples were highly consistent, with no statistically significant differences between the two groups (all *P*>.05; Table 1). The EFA revealed a Kaiser-Meyer-Olkin value of 0.956, and the Bartlett test of sphericity was statistically significant (*χ*²_78_=15,393.4, *P*<.001), confirming the suitability of the data for factor analysis. Principal component analysis suggested a 2-factor solution with eigenvalues greater than 1 (9.42 and 1.26; Figure 1). Factors 1 and 2 explained 72.49% and 9.71% of the total variance, respectively, yielding a cumulative explained variance of 82.2%. Based on the content of the items loading on each factor, factor 1 was labeled “reputation and privacy violation,” as it comprised 9 items (items 5‐13), with factor loadings ranging from 0.9 to 0.947. Factor 2 was labeled “information harassment,” as it consisted of 4 items (items 1‐4), with factor loadings ranging from 0.710 to 0.874 (Table 3).

**Table 3.** Standardized factor loadings of the Social Media Cyberbullying Victimization Scale (n=883).

Items	Factor 1 (reputation and privacy violation)	Factor 2 (information harassment)
1. I have received disturbing messages on social networking sites.	—^a^	0.826
2. I have received insulting messages, comments, or replies on social networking sites.	—	0.874
3. I have been continuously harassed with insulting messages or comments on social networking sites, even after requesting the perpetrator to stop.	—	0.764
4. I have received pornographic images from others on social networking sites.	—	0.710
5. Someone has posted reputation-damaging messages about me on social networking sites.	0.921	—
6. Someone has spread rumors about me on social networking sites to damage my reputation.	0.926	—
7. Someone has discussed me on social networking sites in ways that made my friends dislike me.	0.934	—
8. Someone has discussed matters about me on social networking sites that made me a laughingstock.	0.934	—
9. Someone has hacked my social networking account and posted/sent messages that made me look bad.	0.900	—
10. Someone has shared my secrets on social networking sites.	0.946	—
11. Someone has posted my private/sensitive information online that I didn’t want others to know.	0.947	—
12. I was deceived into sharing my secrets on social networking sites, which were subsequently disseminated.	0.947	—
13. I felt betrayed by friends who posted information about me on social networking sites that I didn’t want others to know.	0.929	—
Proportion of variance explained	72.49%	9.71%
Cumulative variance	—	82.2%

a Not applicable.

#### Confirmatory Factor Analysis

**Figure 2.**
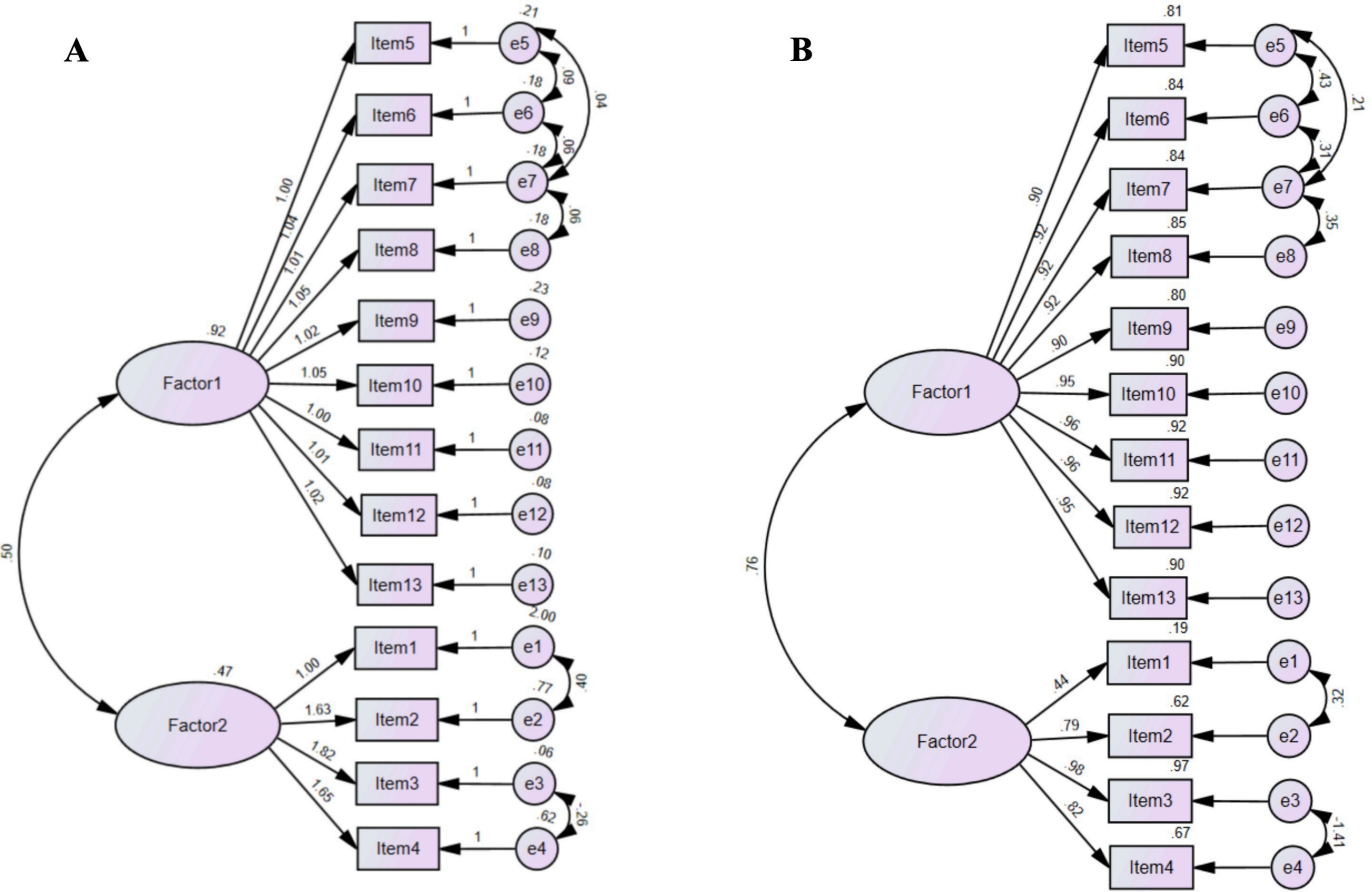
Confirmatory factor analysis of the 13-item Social Media Cyberbullying Victimization Scale (n=883) with unstandardized estimates (A) and standardized estimates (B). The model specifies a 2-factor structure: factor 1 (reputation and privacy violation; items 5-13) and factor 2 (information harassment; items 1-4). Panels A and B present the unstandardized and standardized solutions, respectively, including factor loadings for each item, residual error terms (e1-e13), and their correlated residuals, as well as the covariance between the 2 latent factors.

The CFA path diagram supported a 2-factor structure for the scale (Figure 2). The model fit indices are presented in Table 4. Specifically, the *χ*²/*df* value was 6.0 (*χ*²_58_=347.9), slightly exceeding the reference threshold of <5. However, the other fit indices indicated strong model performance: the RMSEA was 0.075 (<0.08), the SRMR was 0.022 (<0.05), and the GFI (0.943), CFI (0.982), NFI (0.978), TLI (0.975), and IFI (0.982) all exceeded the reference value of 0.9.

**Table 4.** Model fit index of the Social Media Cyberbullying Victimization Scale in confirmatory factor analysis (n=883).

Model fitting index	Reference value	Model value
Chi-square goodness of fit (*χ*^2^/*df*)	<5.0	6.0
Root mean square error of approximation	<0.08	0.075
Standardized root mean square residual	<0.05	0.022
Goodness-of-fit index	>0.9	0.943
Comparative fit index	>0.9	0.982
Normed fit index	>0.9	0.978
Tucker-Lewis index	>0.9	0.975
Incremental fit index	>0.9	0.982

#### Measurement Invariance

Results of the configural, metric, and scalar models across gender and academic majors are available in Table 5. For gender, the configural model showed good fit (*χ*²_116_=503.3, *χ*²/*df*=4.339, CFI=0.976, RMSEA=0.062). Constraining factor loadings (metric invariance) resulted in small changes in fit indices (*χ*²_127_=517.3, *χ*²/*df*=4.073, CFI=0.975, RMSEA=0.059; ΔCFI=−0.001, ΔRMSEA=−0.003), and further constraining item intercepts (scalar invariance) yielded similarly small changes (*χ*²_130_=563.1, *χ*²/*df*=4.332, CFI=0.973, RMSEA=0.061; ΔCFI=−0.002, ΔRMSEA=0.002). For the academic major, the configural model also showed acceptable fit (*χ*²_207_=773.4, *χ*²/*df*=3.736, CFI=0.958, RMSEA=0.056). Imposing metric constraints produced negligible changes (*χ*²_218_=810.2, *χ*²/*df*=3.717, CFI=0.956, RMSEA=0.056; ΔCFI=−0.002, ΔRMSEA=0), whereas the scalar model did not meet the recommended thresholds for invariance (ΔCFI=−0.025).

**Table 5.** Measurement invariance of the Social Media Cyberbullying Victimization Scale (n=883).

Group and model	*χ*²/*df*^a^	CFI^b^	RMSEA^c^	ΔCFI^d^	ΔRMSEA^d^
Gender					
Configural	4.339	0.976	0.062	—^e^	—
Metric	4.073	0.975	0.059	−0.001	−0.003
Scalar	4.332	0.973	0.061	−0.002	0.002
Academic major					
Configural	3.736	0.958	0.056	—	—
Metric	3.717	0.956	0.056	−0.002	0.000
Scalar	5.244	0.931	0.069	−0.025	0.013

a Chi-square goodness of fit.

b CFI: comparative fit index.

c RMSEA: root mean square error of approximation.

d ΔCFI and ΔRMSEA are computed relative to the less constrained preceding model (metric vs configural; scalar vs metric).

e Not applicable.

### Criterion Validity

As shown in Table 6, the SMCVS total score was strongly correlated with its 2 subscales, “reputation and privacy violation” (*r*=0.964, *P*<.01) and “information harassment” (*r*=0.856, *P*<.01). The 2 subscales demonstrated a moderate correlation with each other (*r*=0.688, *P*<.01). In terms of psychological indicators, the SMCVS total score showed moderate positive correlations with depression (*r*=0.484, *P*<.01) and anxiety (*r*=0.389, *P*<.01). Both subscales exhibited similar correlation patterns with these psychological indicators, with correlation coefficients ranging from 0.356 to 0.457 (all *P*<.01).

**Table 6.** Correlations between the Social Media Cyberbullying Victimization Scale scores and psychological well-being indicators (n=1766).

Variables	(1)	(2)	(3)	(4)	(5)
(1) SMCVS^a^ total	1	—^b^	—	—	—
(2) Reputation and privacy violation	0.964^c^	1	—	—	—
(3) Information harassment	0.856^c^	0.688^c^	1	—	—
(4) Depression (PHQ-9^d^)	0.484^c^	0.457^c^	0.433^c^	1	—
(5) Anxiety (GAD-7^e^)	0.389^c^	0.356^c^	0.372^c^	0.733^c^	1

a SMCVS: Social Media Cyberbullying Victimization Scale.

b Not applicable.

c *P*<.01.

d PHQ-9: Patient Health Questionnaire-9.

e GAD-7: Generalized Anxiety Disorder-7.

### Reliability

The internal consistency reliability of the SMCVS, as indicated by Cronbach α, was 0.959, with Cronbach α coefficients for the two subscales of 0.982 and 0.828, respectively. The split-half reliability, measured using the Spearman-Brown coefficient, was 0.973, and the split-half reliability coefficients for the two dimensions were 0.986 and 0.856, respectively. The test-retest reliability of the SMCVS was assessed in 37 medical students with a 3-week interval between measurements. The correlation coefficient between the two measurements was *r*=0.86 (*P*<.001), and the intraclass correlation coefficient was 0.9 (95% CI 0.805‐0.948).

### Receiver Operating Characteristic Analyses

**Figure 3.**
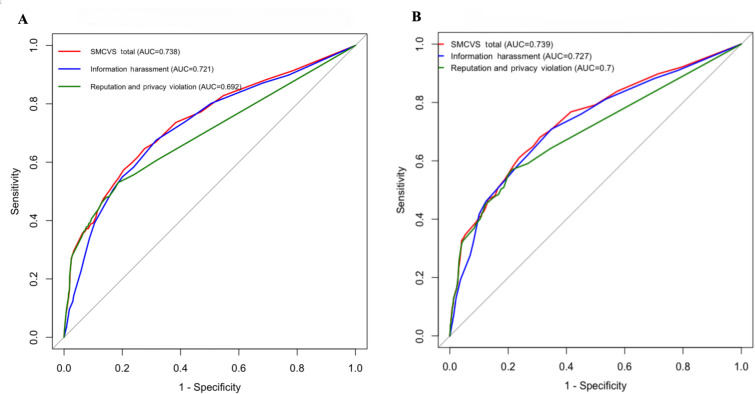
Receiver operating characteristic (ROC) curves for the Social Media Cyberbullying Victimization Scale (SMCVS) total score and its two subscales—“information harassment” and “reputation and privacy violation”—in predicting depression (A) and anxiety (B) among Chinese college students. The curves illustrate the moderate discriminative accuracy of the SMCVS for identifying students at risk of clinically significant depressive and anxiety symptoms.

ROC analyses were conducted to assess the predictive accuracy of the SMCVS and its subscales for depression and anxiety, as shown in Figure 3. For depression screening, the AUC for the SMCVS total score was 0.738 (95% CI 0.709‐0.767), while the AUC values for the “information harassment” and “reputation and privacy violation” subscales were 0.721 (95% CI 0.692‐0.750) and 0.692 (95% CI 0.663‐0.722), respectively, indicating moderate predictive ability. The optimal cutoff score for the SMCVS total score was 19.5, which yielded a sensitivity of 0.646, a specificity of 0.724, LR+ of 2.339, and LR− of 0.489. For anxiety screening, the AUC for the SMCVS total score was 0.739 (95% CI 0.704‐0.775), with the “information harassment” and “reputation and privacy violation” subscales showing AUC values of 0.727 (95% CI 0.691‐0.762) and 0.7 (95% CI 0.663‐0.736), respectively, which also reflected moderate discriminative accuracy. The optimal cutoff score for the SMCVS total score was 22.5, with a sensitivity of 0.610, a specificity of 0.763, LR+ of 2.570, and LR− of 0.511. The AUC values and diagnostic indices for the subscales are also summarized in Table 7.

**Table 7.** Optimal cutoff scores and diagnostic accuracy indices from ROC^a^ analyses of SMCVS^b^ scores for depression and anxiety.

Outcome and predictor	AUC^c^ (95% CI)	Cutoff	Sensitivity	Specificity	LR+^d^	LR−^e^
Depression						
SMCVS total	0.738 (0.709‐0.767)	19.5	0.646	0.724	2.339	0.489
Information harassment	0.721 (0.692‐0.750)	8.5	0.676	0.684	2.136	0.475
Reputation and privacy violation	0.692 (0.663‐0.722)	11.5	0.530	0.814	2.847	0.557
Anxiety						
SMCVS total	0.739 (0.704‐0.775)	22.5	0.610	0.763	2.570	0.511
Information harassment	0.727 (0.691‐0.762)	8.5	0.709	0.651	2.033	0.447
Reputation and privacy violation	0.700 (0.663‐0.736)	11.5	0.571	0.784	2.648	0.547

a ROC: receiver operating characteristic.

b SMCVS: Social Media Cyberbullying Victimization Scale.

c AUC: area under the curve.

d LR+: positive likelihood ratio.

e LR−: negative likelihood ratio.

## Discussion

### Principal Findings

This study adapted and validated a 2D SMCVS for use among Chinese college students. Our findings support the 2-factor structure, comprising “information harassment” and “reputation and privacy violation,” and demonstrate generally sound psychometric properties and meaningful associations with psychological outcomes.

The psychometric properties of the SMCVS support its utility as a measurement tool. The high factor loadings (>0.70) and absence of cross-loadings indicate clear dimensional differentiation. The cumulative variance explained (82.2%) exceeds typical thresholds in scale development research, suggesting comprehensive coverage of the construct [36]. The confirmation of this structure through CFA with satisfactory fit indices further supports the scale’s structural validity. The emergence of 2 distinct dimensions reflects the evolving nature of cyberbullying in the social media era [37]. Information harassment, characterized by direct aggressive communications, represents traditional forms of cyberbullying adapted to social media contexts. Reputation and privacy violation, involving the unauthorized sharing, distortion, or public exposure of personal information and damaging content, captures the unique affordances of social media platforms that enable new forms of victimization targeting both one’s online image and privacy. This dimensional structure advances our understanding of CV by distinguishing between direct aggressive acts and privacy-related violations. In addition, multigroup CFA supported measurement invariance across gender, indicating that factor structure, factor loadings, and item intercepts are comparable and that latent scores can be meaningfully compared between male and female students. For academic major, configural and metric invariance were supported, but scalar invariance was not achieved; therefore, while the structure appears stable across majors, direct mean comparisons of latent scores between different majors should be interpreted with caution, and future studies are needed to further examine and improve cross-major measurement invariance [38].

Some psychometric findings warrant cautious interpretation. Item analysis showed that item 1 had a lower corrected item-total correlation (0.385) than the other items, slightly below the commonly cited cutoff of 0.4 [31]. This item was retained because it captures a broad and relatively mild form of negative experience on social media that may precede or coexist with more explicit cyberbullying behaviors, thereby contributing to content validity. Empirically, item 1 still showed an acceptable loading on its intended factor in the EFA (0.826), and deleting it led to only a negligible change in Cronbach α for the total scale. In addition, although the total and subscale α coefficients were very high (all >0.8), which is often viewed as excellent reliability, such values may also indicate some item redundancy. In particular, the factor loadings for factor 1 were extremely high (0.9‐0.947), suggesting a high degree of homogeneity among the factor 1 items and potential overlap in the information they capture. Future studies could therefore consider removing or rewriting some factor 1 items to reduce redundancy and improve the informational efficiency of the scale. Finally, the CFA yielded a *χ*²/*df* value of 6.0, slightly above the commonly used lenient criterion. This may partly reflect the well-known sensitivity of chi-square to large samples and model complexity [39]; however, given that other fit indices (eg, CFI, TLI, RMSEA, and SRMR) were within acceptable ranges, the overall model fit is considered adequate, while potential local item dependence should be examined in future research.

### Comparison With Prior Work

Scholars have been actively involved in the development of measurement scales for cyberbullying and victimization in recent decades. International measurement scales included the European Cyberbullying Intervention Project Questionnaire, designed for use across 6 European countries [40]; the Cyberbullying Triangulation Questionnaire, created for Spanish students [41]; the Cyber Victimization Experiences and Cyberbullying Behaviors Scales, tailored for British teenagers [42]; the Bullying and Cyberbullying Scale for Adolescents, developed for Australian adolescents [43], and so on [15]. Chinese researchers were also dedicated to developing measurement tools tailored to local culture and national conditions. Lam and Li [16] developed the first scales specifically for CV among Chinese adolescents, known as the E-Victimization Scale. The E-Victimization Scale consisted of only 5 items and was validated as a unidimensional construct. However, the scale was limited to specific social networking platforms, such as email, texting, short messages, and websites like Renren, and focused on a narrow range of cyberbullying behaviors [16]. Rao and colleagues [44] developed a self-designed questionnaire that identified 7 forms of cyberbullying and investigated the prevalence of these 7 types of cyberbullying behaviors among Chinese middle and high school students. Unlike these earlier scales, which primarily targeted adolescents in high school, the scale developed in this study specifically addressed CV among university students. The SMCVS not only removed restrictions on specific social networking platforms but also expanded the scope of behaviors it measured, including harassment, denigration, defamation, sexual harassment, teasing, intrusion, privacy violations, and betrayal. This study conceptualized social media CV as a 2D construct, distinguishing “information harassment” from “reputation and privacy violation.” Although our data do not permit a direct comparison of predictive accuracy between the 2D SMCVS and Lam and Li’s [16] unidimensional model, the differential associations of the two SMCVS dimensions with depression and anxiety suggest a more nuanced characterization of psychological risk. Compared with 7-category behavioral classifications, this 2-factor structure is more parsimonious while still capturing core forms of social media cyberbullying, though future work is needed to examine whether additional, more specific subdimensions further improve the explanation of mental health outcomes.

The differential associations between SMCVS dimensions and psychological outcomes provide important insights into the nature of CV. In contrast to traditional bullying, cyberbullying does not rely on direct interpersonal contact and often provides perpetrators with complete anonymity. The rapid dissemination of harmful messages and images across online platforms can expose victims to a wider audience, intensifying their feelings of vulnerability. Moreover, the pervasive nature of the digital environment makes it significantly harder for victims to evade cyberbullying [44]. Studies have shown that many victims of cyberbullying are also victims of traditional bullying [45]. Psychological health issues among cyber-victims are widespread and may even be more severe than those resulting from face-to-face bullying [46]. The study found an association between CV and depression and anxiety among college students, which was consistent with previous research findings [47,48]. Multivariate logistic regression revealed that participants with depression (odds ratio [OR] 1.40, 95% CI 1.17‐1.67) and anxiety (OR 1.67, 95% CI 1.38‐2.03) were associated with a higher risk of experiencing cybervictimization [48]. General Strain Theory is one of the most commonly applied frameworks for examining the outcomes of cyberbullying [49]. The theory posits that negative interactions or relationships with others—such as being involved in cyberbullying or experiencing CV—can create strain, which may result in adverse emotional reactions (eg, depression or anxiety) and maladaptive coping strategies (eg, substance use, academic underperformance, or deviant behaviors) [50]. The ROC analyses demonstrated acceptable but moderate discriminative accuracy for both depression (AUC=0.738) and anxiety (AUC=0.739), suggesting that the SMCVS may serve as a supplementary screening tool, rather than a standalone diagnostic instrument, for screening students at risk for psychological distress.

### Future Work

Adaptation and validation of the SMCVS specifically tailored for Chinese college students holds significant theoretical and practical value. Given the unique cultural, social, and technological context in China, such a scale can provide a more accurate assessment of cyberbullying experiences and behaviors in this population and may be used in contexts such as academic research, campus mental health services, and policy-related discussions. For instance, it may facilitate the identification of at-risk individuals and the evaluation of prevention and intervention programs, as well as examinations of the prevalence and characteristics of cyberbullying in Chinese higher education institutions. In this context, the ROC analyses provide preliminary support for the screening potential of the SMCVS: the total score showed moderate accuracy in identifying students with depression and anxiety, with optimal cutoff scores of 19.5 and 22.5, respectively, offering a reasonable balance between sensitivity and specificity in this sample and thereby helping to flag students who might benefit from further psychological assessment. Nevertheless, the SMCVS as a screening tool should be used cautiously—higher sensitivity may increase false positives, whereas higher specificity may increase false negatives, meaning that some distressed students could still be missed. Moreover, these cutoff points are sample- and prevalence-dependent estimates derived from cross-sectional data and should not be regarded as definitive; their performance and practical implications need to be re-evaluated in settings with different base rates of depression and anxiety and, ideally, within prospective designs. In clinical and service settings, a 2-stage strategy may be preferable, whereby the SMCVS is used for initial screening followed by more comprehensive clinical or psychological assessment for individuals who screen positive.

### Limitations

Several limitations should be noted. First, the cross-sectional design of this study precludes causal inferences about the relationships between CV and psychological outcomes. Longitudinal studies are needed to establish the directionality and underlying mechanisms of these associations. Second, the study was limited to Chinese college students, and the SMCVS was adapted and validated within the mainland Chinese social media ecosystem, where platforms such as WeChat, QQ, and Xiaohongshu are predominant, which may restrict the generalizability of the findings to other cultural contexts. As patterns of use and platform features may differ across social media environments, the applicability of the SMCVS to other platforms and cultural contexts remains to be further examined. Cross-cultural and cross-platform validation studies including participants from more varied backgrounds would help enhance the scale’s utility and the universality of the results. Third, the reliance on self-report measures may introduce recall bias, as participants may not accurately remember past experiences, and social desirability effects, where they may underreport or overreport certain behaviors to present themselves in a favorable light. Using multimethod assessments, such as peer reports or observational data, could help address these issues. Fourth, although this study did evaluate the test-retest reliability of the SMCVS, this analysis was conducted in a relatively small subsample of 37 medical students. The high correlation coefficient therefore provides only preliminary evidence regarding the temporal stability of the scale and may not be fully representative of the broader college student population or different academic majors. Future research should replicate the test-retest assessment in larger and more diverse samples to further confirm the robustness and generalizability of the scale’s stability over time.

### Conclusions

This study establishes the SMCVS as a psychometrically sound and culturally relevant instrument for assessing social media cyberbullying victimization among Chinese college students. By validating its 2D structure, this research captures the specific manifestations of cyberbullying within the contemporary Chinese social media ecosystem. Consequently, it provides researchers with a reliable tool to track victimization trends and evaluate the effectiveness of future preventive programs. Furthermore, the significant associations found between CV and adverse psychological outcomes highlight an urgent need for action. These findings strongly advocate for the integration of targeted cyberbullying screening and coping strategies into university mental health initiatives, ultimately fostering a safer digital environment and supporting the overall psychological well-being of young adults.

## Abbreviations

AUC: area under the curve
CFA: confirmatory factor analysis
CFI: comparative fit index
CV: cyberbullying victimization
EFA: exploratory factor analysis
GAD-7: Generalized Anxiety Disorder-7
GFI: goodness-of-fit index
I-CVI: item-level content validity indices
IFI: incremental fit index
LR+: positive likelihood ratio
LR−: negative likelihood ratio
NFI: normed fit index
OR: odds ratio
PHQ-9: Patient Health Questionnaire-9
RMSEA: root mean square error of approximation
ROC: receiver operating characteristic
S-CVI/Ave: scale-level content validity index/average
SMCVS: Social Media Cyberbullying Victimization Scale
SRMR: standardized root mean square residual
TLI: Tucker-Lewis index
*χ*2/*df*: chi-square goodness of fit
